# Bridging approaches to facilitate innovation: building an approach for heated tobacco products from case studies in the food and drug domains a comparative review

**DOI:** 10.1007/s00204-025-04081-5

**Published:** 2025-05-21

**Authors:** Ruth Dempsey, Irfan Gunduz, Patrick Vanscheeuwijck

**Affiliations:** 1 Science Speaks Consultancy Sàrl, Le Mont Sur Lausanne, Switzerland; 2 PMI R&D, Philip Morris Products S.A, Quai Jeanrenaud 5, CH-2000 Neuchâtel, Switzerland

**Keywords:** Heated tobacco products, Innovation, Risk assessment, Bridging

## Abstract

An increasing number of heated tobacco products (HTPs) have been commercialized in several countries over the last decade. To benefit public health, these products should have a lower health risk profile than cigarettes. This includes the need to be sufficiently acceptable to adults who smoke so that a reasonable proportion of those who do not want to quit smoking are persuaded to switch completely. Additionally, the product should not increase the likelihood of non-smokers starting to use tobacco or smokers increasing their total consumption of tobacco products. Part of this process involves iterative development of new product versions, leveraging lessons learned from consumer experiences with marketed products, and applying novel technologies to improve the consumer product offering. Risk assessment for these products may include pre-clinical quality, analytical and toxicity evaluations, pre-market clinical studies, and post-market surveillance studies. In recent years, approaches to bridge the assessment for modified or new products to data generated for reference products by comparing their equivalence were proposed. Here, we review the approaches taken for such bridging studies and relate them to product comparability and bridging approaches established in a diverse range of consumer and pharmaceutical products. This leads to a proposal for a logical, stepwise, and tiered bridging approach to effectively manage the introduction of new HTPs through scientific substantiation and have potential to increase the public health benefit by reducing risk and improving product acceptability for adult smokers without attracting non-smokers by new innovations.

## Highlights

- Product innovation could improve the harm reduction potential of heated tobacco products (HTPs).
- Extensive risk assessment requirements may stifle product innovation or unnecessarily slow the time to market for new products.
- Establishing critical drivers of a product’s toxicological risk profile can be used to bridge to a reference product’s assessment profile.
- The principle of bridging new products to fully marketed predicate products based on a reduced testing schedule is well-established in many fields.
- Similarities in the approach used for dietary supplements, nutraceuticals, and biosimilars provide a basis for a bridging approach for HTPs.

## Introduction

Although global smoking prevalence has declined in recent decades, more than 1 billion people still smoke (Global Burden of Disease Tobacco Collaborators 2021). Exposure to harmful and potentially harmful constituents (HPHCs) produced during tobacco combustion is the primary cause of smoking-related diseases (Saha et al 2007). While cessation is the best option to reduce the risk of smoking-related diseases, smokers switching from combusted to non-combusted products could play a complementary role in tobacco harm reduction (Hatsukami and Carroll 2020). Heated tobacco products (HTPs) were developed with the aim of reducing the harm caused by smoking by presenting an acceptable alternative for adults who would otherwise continue to smoke. On a population basis, the potential benefits of smokers switching to such products will be maximized by encouraging more smokers to switch. Smokers have a relatively broad range of combustible products, such as cigarettes, available to them and base their choice on several different reasons, including perceived strength, taste, smell, and visual appearance (Cowie et al 2014; Fagerström and Bridgman 2013).

Although HTPs have been relatively successful in some markets for several years, not all adults who smoke find that these alternatives meet their expectations to the extent that they are willing to quit cigarettes. In Japan, possibly the most advanced alternative tobacco product market, 18.5% of the population used tobacco or nicotine-containing products in 2017, of which 1.8% had completely switched to HTPs. In addition, 20.6% of HTP users reported dual use (Afolalu et al 2022). The number of complete switchers may increase if adults who smoke are offered a broader range of products to match a wider range of tastes and expectations.

HTPs rely on various technologies to heat tobacco in a controlled manner, without combustion, and as consistently as possible. The technology of heating tobacco is continually developing, with newer versions providing additional advantages (such as reduced reliance on battery power) and an improved consumer experience (for example, more uniform heating, ease of use, and closer sensory similarity to cigarettes).

Alterations to the tobacco matrix, such as adding or using materials other than tobacco, using tobacco from different sources, or treating or processing the tobacco differently to adapt and function optimally with new technologies, may also improve the consumer experience.

It is important to establish that any changes made to a product do not adversely affect the expected risk reduction potential compared to cigarettes. In part, this can be achieved by ensuring that the mechanism of aerosol production without combustion is maintained as previous assessments have indicated that this is the driver to achieve reduced emissions and exposure to harmful constituents (Mallock et al 2019; Schaller et al 2016).

Performing a complete risk assessment for any product is time-consuming and may be seen as an unnecessary hurdle for product innovation. Determining how much repeat assessment is required will depend on the degree of change of the innovative product compared to the reference product. A bridging protocol can help determine, in a stepwise and logical manner, how to evaluate differences between products and decide what level of testing may be required for different types of product innovations. Appropriately designed bridging assessments can thereby facilitate product innovation and shorten the time from prototype to market.

To date, we are not aware of any regulatory authority that specifies requirements for bridging assessments for innovation of an authorized HTP although there are several publications that discuss the context of bridging for HTPs. This is perhaps not surprising, given that the HTP market is relatively new, and many countries are still in the early stages of establishing regulatory oversight. It may be useful, therefore, to learn from how regulatory authorities have looked at similar bridging approaches for other regulated products.

The purpose of this paper is to review how bridging risk assessments for evolutions of consumer products are currently performed, both for HTPs and in other contexts, from which an optimized bridging assessment approach can be derived to expedite product innovation in the HTP category.

Tobacco products present specific challenges and are regulated differently depending on the country. The specifics of the risk assessment required to support new product launches also vary across countries (Institute for Global Tobacco Control 2020). This presents a challenge in identifying other categories that the bridging approach can be usefully compared against. The categories of products for which bridging approaches were reviewed in this paper were selected because they have regulatory oversight, are consumer goods that offer health or wellness benefits linked to harm reduction, and can evolve with new technologies.

### Definitions: bridging and comparability

For the purpose of this review, we use the terms comparability testing and bridging to refer to two different aspects of comparison for product innovation in a specific context. In general agreement with the concepts applied in the context of health claims or harm reduction claims, the following definitions are applied:

1. Comparability testing may be used to assess the equivalence of two product formulations, such as new tobacco blends in HTP consumables or two devices using the same consumable. The purpose is to ensure that the aerosol produced and inhaled under normal use conditions for new formulations or devices are comparable to that of a reference product. As a result, health claims related to the reference product may be considered equally applicable to a new product that meets the standard of equivalence through comparability testing.
2. Bridging studies are conducted to establish a link between existing scientific evidence on a reference product and a subset of assessments on a new product or formulation. For example, for an authorized health claim to be considered applicable to a product similar to one with existing scientific evidence, bridging studies can demonstrate that the new product is equivalent to the reference product to the extent that the existing evidence on the reference product is also relevant for the new product. This may apply even in the case of significant changes in the technology or design of the new product if the key driving parameters for the health claim are maintained. The objective of bridging testing is to ensure there is sufficient data to be confident that the extensive scientific data from the reference product is also relevant and valid for the new product.

### Bridging and comparability assessment of heated tobacco products

**Fig. 1:**
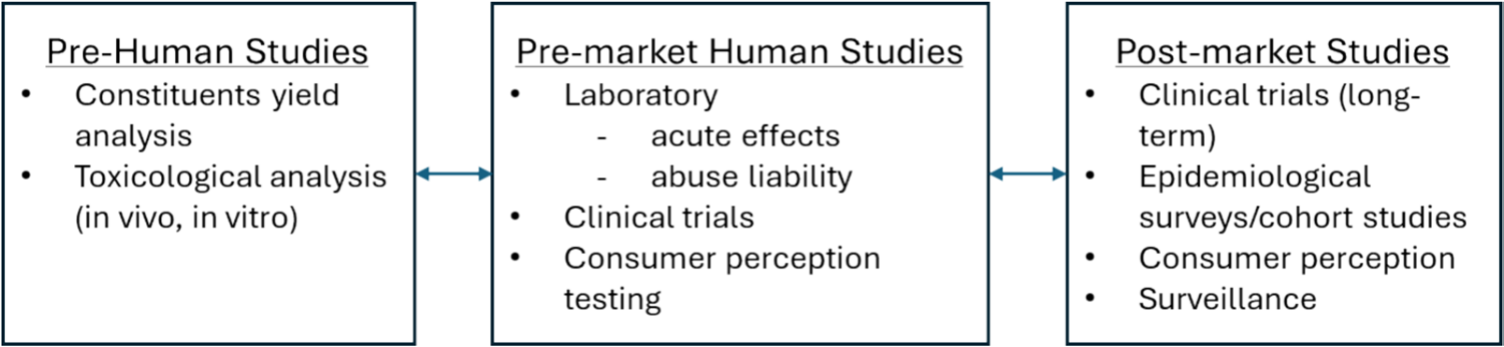
Outline of assessment requirements for modified risk tobacco products, from Hatsukami et al. 2012 (Hatsukami et al 2012)

HTPs were developed on the principle of producing an aerosol from heating rather than burning tobacco. Compared to cigarettes, HTPs produce an aerosol containing substantially lower levels of HPHCs (Mallock et al 2018, 2019; Sussman et al 2023). HTP risk assessments covered a comprehensive range of toxicological assessments, including chemical and physical characterization of the aerosol, in vitro and in vivo studies, clinical studies to measure the exposure and biomarkers of effect in human subjects (Smith et al 2016), and perception and behavior studies to evaluate how the presented product may impact consumer consumption of tobacco products overall (Fig. 1) (Hatsukami et al 2012; Smith et al 2016).

The results of these assessments across a broad range of HTPs have consistently demonstrated reductions in the HPHCs of the aerosol from the HTPs in comparison to the smoke from cigarettes across this hierarchy of assessment (Boué et al 2019; Malt et al 2022).

Design features can reliably predict the testing outcome based on the previous knowledge and data that has been accumulated from similar products. This can reduce the need for extensive testing of the product. In the case of HTPs, for example, the design feature of heating tobacco instead of burning it, thereby eliminating the process of combustion and the chemicals formed during combustion, has been repeatedly demonstrated to result in a reduced level of harmful components in the aerosol in comparison to the smoke from cigarettes (Baker 2006; Lang et al 2024; Schaller et al 2016). Consequently, complete characterization of the aerosol for new product variants may not be necessary. Carrying out extensive assessments may waste resources since the overall aerosol chemistry can be reliably predicted based on establishing the principle of heating the tobacco without combustion. Confirmation of an absence of combustion could be verified by measurement of certain constituents known to be formed and emitted at elevated levels during combustion, such as CO, NO, or NO_x_, which are specifically produced at high levels during the high-temperature oxidative processes of combustion (Cozzani et al 2020).

Theoretically, this assumption could be extended across the entire range of product assessments as these assessments can be considered to be a causal chain of events, whereby the reduction in key elements in the aerosol leads to lower in vitro and in vivo toxicity, which in turn results in reductions in biomarkers of exposure and effect in clinical studies (Schlage et al 2020; Smith et al 2016). Thus, theoretically, it could be assumed that any new product variant that demonstrates heating without combustion could be confidently assumed to have a similar performance without the need for the complete range of assessments that were relied upon for marketing authorizations of previous comparator products. However, there may be other considerations such as the influence modifications may have on the behavior of the consumer that will need to be evaluated to ensure appropriate confidence in the applicability of the previous comparator product assessments to the new product.

**Fig. 2:**
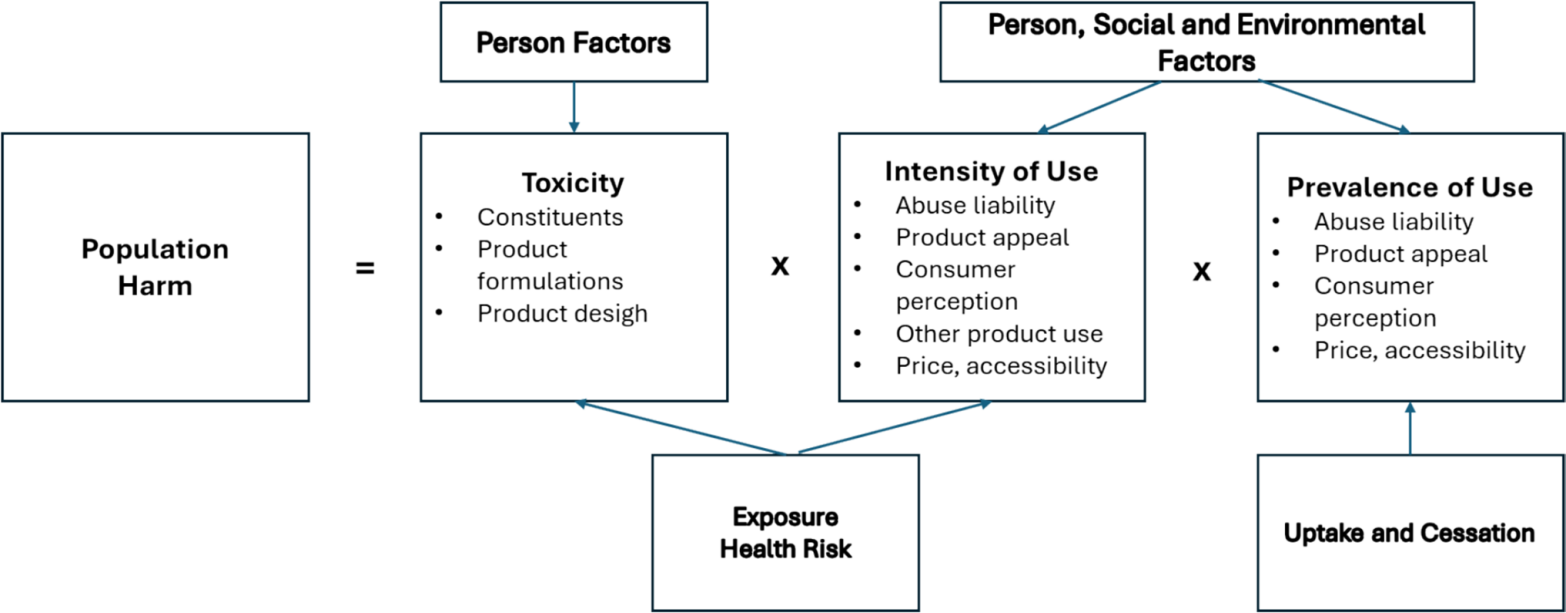
Components to assess population harm, from Hatsukami et al. 2012 (Hatsukami et al 2012)

Confidence in the applicability of the data from the reference product may be increased by applying a limited range of additional assessments on the new product to verify that it is performing comparably on a subset of data. In the specific case of tobacco products, one of the challenges in bridging assessments is the consideration of the potential for product misuse or abuse. As illustrated in Fig. 2, the potential for population harm reduction is related to both a reduction in toxicity alongside having a sufficiently acceptable product to encourage adult smokers to switch from the more harmful products, while assuring that the modified product does not increase overall tobacco use (Hatsukami et al 2012). This creates a particular challenge for the evaluation of product innovations, where the balance of making the product more acceptable to smokers has to be constantly weighed against the potential to unintentionally attract non- or ex-smokers.

There is no specific guidance on abuse liability when testing tobacco products. Research on the abuse liability of cigarettes and other nicotine products shows that the abuse liability is multifactorial and may be influenced by many aspects of the product’s design (Henningfield et al 2011).

However, several approaches have been used, including pharmacokinetic studies that analyze blood nicotine levels following product use, subjective and behavioral responses, and/or physiological responses. Such studies have generally followed similar designs, which are aimed at producing as near as possible real-life conditions in a pre-market environment following the recommendations of Vansickel (Vansickel et al 2021). Following an introduction to the product, adult smokers are selected based on their interest in trying the product. After a period (usually about a week) where baseline tobacco use details are recorded, subjects are offered free access to the test product for a period of around 6 weeks. Subjects are free to use the new product or revert to their original products throughout the study. Detailed diaries are kept during the study to record all tobacco and/or nicotine product use as well as subjective analysis of their product experience. The key measurements for these studies are the number of test products that each subject is using at the end of the study period, the overall number of tobacco and nicotine products used per day (which should not be greater and has usually been found to be less than baseline), and the reduction in the number of cigarettes or combustible products used over the study period. Subjects’ enjoyment of the new products, along with expressing a willingness to switch, is also recorded (Roulet et al 2019; Shetty et al 2022; Vansickel et al 2021).

The increasing number of alternative tobacco products coming to market in recent years has allowed further research into the links between the pharmacokinetics of nicotine, other aspects of product design (such as flavor), and actual product use behavior as measured through post-marketing surveillance. A repeated finding of these studies is that offering a wide variety of designs, such as different levels of nicotine or different flavors, appears to encourage more people to adopt the new products and facilitate a larger proportion of full switching from cigarettes (Campbell et al 2022; Hajek et al 2017; McCaffrey et al 2022; Shiffman et al 2021).

#### Regulations on comparability for tobacco products

With respect to tobacco products, in the US, the Food and Drug Administration (FDA) has the authority to evaluate and approve modifications to tobacco products that have been granted a modified risk tobacco product (MRTP) authorization. The proposed modifications must be compatible with the MRTP order and may require additional data or studies to support the modification. Though specific guidance on what might be required for MRTP modifications is limited, the FDA’s guidance on substantial equivalence requires side-by-side quantitative and qualitative comparisons of the new tobacco product with the predicate tobacco product concerning all product characteristics, including HPHC emissions and the functioning of the heating source (FDA 2011). The FDA will issue an order of substantial equivalence if the new product (1) has the same characteristics as the predicate product, or (2) has different characteristics but submitted information showing that it does not raise different public health questions (FDA 2021).The FDA’s substantial equivalence requirement serves as a bridging approach for new version of products that were marketed before February 15, 2007. However, this requirement does not apply to heated tobacco products that were introduced after the cut-off date.

In the EU, HTPs are regulated as novel tobacco products according to the EU Tobacco Product Directive, for which member states have some flexibility on how to implement the regulation of such products (European Union 2014). Member states require notification of any novel product 6 months prior to launch, which includes available scientific studies, but no formal process for product changes is described.

#### HTP comparability approaches in the literature

Several proposals for bridging strategies have appeared in scientific literature recently. Schlage et al. (2020) present a “bridging use case” comparing non-clinical evidence from two HTPs, to systematically follow the consequences of reduced aerosol chemistry and toxicity through the causal chain of events linking smoking to disease (CELSD), using systems biology and toxicology to extrapolate to human studies (Schlage et al 2020). In this case, the two HTP products use different approaches to heat the tobacco, one through electrical heating (THS2.2) and the other using a carbon heat source to heat a tobacco plug (CHTP1.2). Although quite different in heating technology, both products were found to have similarly reduced emissions of selected HPHCs relative to cigarettes, leading to similar reductions in HPHC exposure and uptake in in vivo inhalation studies, in cytotoxic and mutagenic potential in vitro assays, and in effects on human organotypic nasal epithelium in vitro*. *In vivo studies in rats and mice also showed similar reductions for both products in effects in the nasal epithelium, lung inflammatory processes, molecular effects in the lungs, lung disease-associated endpoints, acceleration of atherosclerotic plaque formation, gene expression effects in the heart, similar effects in the liver, and these similarities were also found in testing protocols that simulated the effects of switching to these products compared to complete cessation. They were thus able to demonstrate similarity in reduced exposure and risk profiles for both products across the CELSD (Schlage et al 2020).

Similarly, Gaca et al. (2022) published a rationale for reduced testing of product variants, with proposed criteria for establishing changes that could be considered minor, with minimal requirements for testing, as opposed to changes that might require more extensive testing (Gaca et al 2022). In a later publication, Goodall et al. (2022) compared HTPs that differed in tobacco composition and devices with similar forms of heating with different temperature profiles. Targeted aerosol chemistry from all of these products was found to be comparable, indicating consistent reductions of above 99% for analytes that are formed and emitted at high levels during combustion (e.g., CO) and that are generally found to be low and often, with the current analytical methods, below the limit of quantification for HTPs and similarly reductions of greater than 90% for selected toxicants expected to be present at much lower levels than from combustible products. Based on these comparisons, the authors conclude that there is sufficient data to show that different iterations of HTPs maintain substantially reduced toxicant emissions and consequent reductions in measured aspects of biological activity in comparison to cigarettes (Goodall et al 2022).

### Comparison to product innovation assessment approaches in other industries

Similar situations arise in many different regulated product environments, and it may be helpful to compare how comparability and bridging approaches are applied to innovations of products with health or harm reduction claims.

For example, the FDA process for approving generic drugs is designed to ensure that the generic product is safe, effective, and equivalent to the pre-approved brand-name drugs they reference. The generic drug application must include studies designed to demonstrate that the products meet the same standards of quality, strength, purity, and stability as the original reference product. They must also show that their products have the same active ingredient, dosage form, route of administration, and labeling as the reference product (FDA 2019). However, there is a difference between the active ingredient comparator for pharmaceuticals and potential comparators for HTPs. Tobacco-based products are present as complex mixtures rather than as simple ingredients. A more informative comparison may be made with biological therapeutics (biologicals and biosimilars) or dietary supplements, nutraceuticals, and functional foods.

Biologic therapies are medicinal products made by or derived from the living cells of humans, animals, or microorganisms. Examples include vaccines, blood products, cytokines, and monoclonal antibodies. Biosimilars are biologic therapies approved for marketing on the basis of data demonstrating that they have physicochemical and functional characteristics comparable to those of a previously licensed biologic therapy. Although not strictly complex mixtures, biologics are complex molecules (Chao et al 2015).

Dietary supplements are generally recognized to include products intended to supplement the diet and containing either vitamins, minerals, herbs or botanicals, amino acids, a dietary substance that increases the total dietary intake, or a concentrate, metabolite, constituent, extract or combination of these substances, and that are taken in a dosed form (e.g., pills, tablets, soft gels, capsules, liquids in measured doses, etc.). Functional foods or nutraceuticals have health benefits beyond their basic nutritional value, including isolated nutrients or non-nutrient substances with a physiological effect (Djaoudene et al 2023).

Factors, such as rising healthcare costs, increasing consumer awareness, and focus on changing lifestyles, have resulted in the dietary supplement and functional food market experiencing significant growth worldwide over the past decade, and this growth is expected to continue significantly in the coming decades. Rising demands for consumer products diversified across different types, forms, health applications, and distribution channels, including a growing preference for herbal, vegan, and probiotic products, and trends in personalization and digitalization are encouraging further product innovation in this field (Djaoudene et al 2023).

Concerns regarding potentially misleading health claims and the impact of health claims on perception and behavior for these nutritional products reflect similar concerns for harm reduction in the tobacco industry (Ashwell et al 2023). For example, the preamble to regulation (EC) 1924/2006 for health claims on foods highlights concerns around perceived advantages of foods with health claims encouraging consumers to “make choices which directly influence their total intake of individual nutrients or other substances in a way which would run counter to scientific advice” (European Union 2006).

#### Regulations on biologicals and biosimilars

Although there are only a few examples of complex mixtures in product regulations, there are lessons to be learned from the treatment of biosimilars. The complexity of the production means that biosimilars are not exact replicas of the reference product. For biosimilars to gain approval in the EU or the US, the similarity in terms of quality, biological activity, safety, and efficacy must be demonstrated. If achieved, the demonstration of similarity allows for an abbreviated series of non-clinical and clinical assessments as the safety and efficacy data from the reference biological can be relied upon (Declerck and Farouk Rezk 2017; Smeeding et al 2019).

#### Regulations on dietary supplements

Dietary supplement regulations are not harmonized internationally and thus differ considerably in their application. The key objectives for regulatory oversight of dietary supplements are to ensure quality, safety, and efficacy. In contrast to pharmaceutical products, regulations are based on monitoring the market for safety and proper labeling compliance, while placing more responsibility on manufacturers than on pre-market approval (Dwyer et al 2018).

In Europe, nutritional or health claims are regulated by the European Food Safety Authority (EFSA) as pertaining to food products. The EFSA evaluates safety and bioavailability of nutrient sources and authorizes health claims. Health claims for these types of products in the EU are regulated under the regulation EC No 1924/2006 on nutrition and health claims made for foods (European Union 2006), and must be authorized following scientific assessment by EFSA, based on guidance provided by the EFSA Panel on Nutrition, Novel Foods and Food Allergens (NDA Panel) (EFSA 2023). The regulation also recognizes that health claims based on generally accepted scientific evidence should be permitted based on a community list of such permitted claims after consultation with the EFSA. This means that manufacturers may directly apply a health claim that appears on the permitted list provided that they establish the product meets the food and nutrient criteria relevant to that claim without the necessity for further scientific verification. By implication, bridging or comparison to other products on the market is established by demonstrating that the product meets the characterization criteria according to established measurement methods (e.g., analysis of ascorbic acid levels for vitamin C). Interestingly, the regulation also recognizes an accelerated type of authorization for health claims based on newly developed scientific evidence to stimulate innovation (EFSA 2023).

In the US, dietary supplements are regulated by the FDA as food products. They do not need to be approved by the FDA before they are marketed, and the FDA does not evaluate the safety or effectiveness of dietary supplements. However, they will take action against any product on the market that is found to be adulterated, misbranded or makes false or misleading claims. Thus, the manufacturer must ensure that all products on the market comply with the Federal Food, Drug, and Cosmetic Act and other relevant laws. The Federal Trade Commission is responsible for enforcing the truthfulness and the accuracy in advertising for dietary supplements and other health-related products.

Although post-market surveillance requirements do not appear to be specifically required for dietary supplements, there is an implied responsibility for manufacturers to maintain awareness of any relevant development in the science, and the regulators can and do monitor the market and have the power to remove the authorization of any products in light of new information.

*Case study 1* Dietary supplements claims on the maintenance of functions of the immune system

Several vitamins and essential minerals positively affect the immune system and support the body’s natural defense functions through various mechanisms. In Europe, in 2009, the EFSA NDA panel approved claims on the maintenance of the immune system based on the essentiality of vitamins C, D, A, B12, B6, Zinc, Copper, Folate, Iron, and Selenium (EFSA 2016). With these approvals, manufacturers of dietary supplements may apply these claims based on an application that demonstrates that the food or constituent and its physical and chemical characteristics, manufacturing processes, stability, and bioavailability meet the requirements for the relevant vitamin or mineral on this list.

In the case of vitamin C, for example, an application to EFSA was made to substantiate health claims related to vitamin C and protection of DNA, proteins, and lipids from oxidative damage, antioxidant function of lutein, maintenance of vision, collagen formation, function of the nervous system, function of the immune system, function of the immune system during and after extreme physical exercise, non-heme iron absorption, energy-yielding metabolism and relief in case of irritation of the upper respiratory tract (EFSA 2009a). The process for authorization of these claims involves:

1. Characterization of the food constituent: It was concluded that vitamin C is well-characterized and measurable in foods by established methods.
2. Relevance of the claimed effects on human health: This is based on a review of the science relating to each of the claims to establish if the effect is associated with a health benefit. For example, for the protection of DNA, proteins, and lipids from oxidative damage, the panel agreed that oxidative damage caused by free radicals is detrimental to cellular function and, therefore, agreed that protection of DNA, proteins, and lipids from oxidative damage would be beneficial to human health.
3. Scientific substantiation of claimed effect for the specific food element (in this case, vitamin C): For each aspect of the claim, the scientific panel reviewed the literature and any other studies submitted concerning the effect and concluded that the evidence was sufficient to establish a cause-and-effect relationship between dietary intake of vitamin C for all of the elements of the claim except the promotion of the antioxidant function of lutein, maintenance of normal vision, and relief in case of irritation in the upper respiratory tract, which were consequently not authorized as health claims for vitamin C.
4. Wording of the claim: The review of the wording of the claim is directed at ensuring that it is scientifically supportable and neither inaccurate nor misleading.
5. Finally, conditions and restrictions under which these claims can be applied are defined. In the case of vitamin C, the conditions for the approved claims were that the food should be a source of vitamin C. Concerning the function of the immune system during and after extreme physical exercise, the food should contain at least 200 mg vitamin C to be consumed daily in addition to the usual diet.

However, claims other than those based on the essentiality of nutrients, such as specific aspects of immune system function, have not been approved. Manufacturers wishing to make specific claims would be required to provide scientific substantiation, including studies on changes in immune markers or functionality (e.g., inflammation), along with evidence of a beneficial physiological effect or clinical outcome (EFSA 2016).

*Case Study 2* Plant stanols and claims on lowering blood cholesterol

Plant stanols are naturally occurring substances that can lower blood cholesterol levels by reducing the absorption of dietary cholesterol in the intestine (Poli et al 2021).

In the late 1990 s, after several clinical trials demonstrated their safety and efficacy, manufacturers started adding plant stanols to some functional food. The first food items which were granted to make claims about their plant stanol-based cholesterol lowering properties were margarines. Over the years, the range of products enriched with plant stanols and sterols has expanded to include yogurts, milk, juices, and cereal products (Barkas et al 2023). Concerns around global increases in elevated total cholesterol have led to guidelines that recommend lifestyle changes including functional foods as preventative measures before pharmaceutical interventions (Mach et al 2019). A recent increase in the popularity of plant stanols in functional foods can be attributed to the scientific evidence of their beneficial effects on cardiovascular health and the growing consumer demand for alternatives to the pharmaceutical lipid-lowering statins that can help lower cholesterol levels without the perceived negative side effects (Osadnik et al 2022; Poli et al 2021). As new plant stanol-enriched products launched into the market, it is, of course, important to ensure that they are effective in the management of cholesterol levels. Thus, a bridging strategy for such products is required.

In a similar process to that described for dietary supplements above, the EU NDA expert panel reviewed several health claims for plant sterols/stanols (EFSA 2008, 2009b; EFSA 2010):

1. Characterization of the food constituent: The panel agreed that the term plant sterols and plant stanol esters are well-characterized.
2. Relevance of the claimed effect on human health: Based on the established association between blood cholesterol levels and cardiovascular health, the panel concluded that maintaining normal blood cholesterol concentrations is a beneficial physiological effect.
3. Scientific substantiation of the claimed effect: The expert panel concluded that a clinically significant LDL cholesterol lowering effect of between 7% and 10.5% could be expected by a daily intake of 1.5–2.4 g of plant sterols/plant stanols in an appropriate food matrix has been established.
4. Wording of the claim: The approved wording was: “Plant sterols/stanols contribute to the maintenance of normal blood cholesterol levels.”
5. Conditions and possible restrictions of use: To bear the claim, a food should provide at least 0.8 g per day of plant sterols/stanols in one or more servings. The food matrix is restricted to foods, such as margarine-type spreads, mayonnaise, salad dressings, and dairy products, such as milk, yoghurts, and cheese. The efficacy of plant sterols/stanols added to other food formats is considered less well-established.

### Proposed bridging protocol for HTPs

It is likely that the variety of HTPs offered to consumers will increase in accordance with their popularity and success in displacing the more harmful, combusted counterparts. It therefore seems timely to consider approaches to effectively evaluate new products. The examples from the world of dietary supplements and functional foods reviewed above may not be perfect comparisons, but they do have sufficient in common with a growing HTP market to provide some useful guidance in how HTP variants could be assessed. Below we outline an effective set of assessments that can characterize a new product sufficiently to be assured that it qualifies as an HTP and that the generally accepted attributes of HTPs as authorized for marketing will also apply to the new product innovation. This includes providing evidence that differences in the product from an already substantiated reference product would not be expected to add new or increased toxicological concern, either through unintentional changes to product performance or have an increased potential for abuse liability.

Such a bridging protocol is proposed to include the following steps:

1. Characterization

The starting point of a bridging assessment should be to characterize the new product. This is a relatively simple process for the vitamin C and plant stanols discussed above, as the key active feature of each is well defined and there are established analytical methods to characterize them. The complex nature of both the biological tobacco matrix and the aerosol produced from an HTP make for a less straightforward approach. As with biosimilars, the similarity needs to be established by looking at key aspects of the products. For HTPs, the key factor is the creation of the inhalable aerosol by heating a tobacco matrix in a controlled manner that ensures an absence of combustion and a consistency of the aerosol. This may be achieved in different ways. Thus, the first step in a bridging assessment should be a full description of product design features that are compatible with controlled heating where no combustion of the heated substrate occurs. Clearly at this stage, a toxicological assessment of all the ingredients and materials used in the new product design should also be performed and individual risk assessments should be conducted for the new features.

2. Scientific substantiation of product claim or category

The EFSA approach for dietary supplements described above first establishes a claim based on scientific substantiation that can then be applied to any product that meets the characterization and conditions determined as relevant for that claim. In a similar way, HTPs as a category have been established as appropriate alternatives to combustible cigarettes in several markets. The second stage in a bridging assessment can be seen as an extension of the characterization of the products design features, to ensure that the product performs sufficiently similarly to the reference product for the product status to be applicable to the new product.

As reviewed in this paper, the scientific basis for the HTP category of products has been established based on extensive analyses consistently showing that compared to combusted cigarettes, HTPs produce lower levels of HPHCs in the aerosol. The relevance of these reductions in HPHCs has been confirmed through HTP risk assessments including toxicological assessment, in vitro and in vivo studies, clinical studies and perception and behavior studies (Hatsukami et al 2012; Mallock et al 2018, 2019; Smith et al 2016; Sussman et al 2023). In the context of bridging, therefore, it should be possible to establish that a new HTP product variant meets the appropriate category standard by demonstrating that this reduction in HPHCs is maintained with the new product design.

It is therefore proposed that the second stage of this bridging assessment should be to perform aerosol chemistry evaluations demonstrating lack of combustion and reductions in key HPHCs (such as the World Health Organization (WHO)−9 list of constituents (WHO 2008) to validate aerosol production is consistent with the reductions expected for an HTP product category.

The absence of combustion is a critical indicator for substantial reduction of HPHCs in HTP aerosol compared to cigarette smoke. A recent market survey examined eleven products from various markets, confirming the absence of combustion based on British Standards Institute Publicly Available Specifications (BSI PAS) 8850:2020 standards.These products feature different heating profiles, consumable designs, and blends, with the common factor being that they produce aerosol by heating tobacco rather than burning it. The study's findings show that the aerosol from all these products averages over a 92 percent reduction in HPHCs, as defined by several regulatory bodies including the WHO's 9 priority toxicants recommended for mandated reduction in cigarette smoke (WHO 2008), the FDA's 18 constituents required for reporting in cigarette smokes (FDA 2012b), and the WHO's 39 constituents non-exhaustive list of priority toxicants recommended for monitoring (WHO 2015), compared to the levels found in reference cigarette smoke (Gunduz 2024). Differences in the levels of reduction are primarily influenced by tobacco-dependent constituents, such as ammonia, tobacco-specific nitrosamines, or constituents formed from precursors at low temperatures (Gunduz 2024). Among these, the WHO-9 list of constituents including CO, acetaldehyde, formaldehyde, acrolein, B[a]P, benzene, 1,3-butadiene, NNN, and NNK can be quantified for bridging studies for the following reasons:

- This concise list includes priority toxicants from various chemical classes, such as volatile organic compounds, gasses, polycyclic aromatic hydrocarbons, aldehydes, and tobacco-specific nitrosamines in cigarette smoke.
- WHO Tobacco Laboratory Network (TobLabNet) has established methods to measure these constituents placed in the WHO-9 list in mainstream cigarette smoke (WHO TobLabNet SOP 9 2018), and also ISO 17025-accredited methods are developed to test them in HTP aerosol.
- They can serve to estimate the reduction of HPHCs in HTPs compared to cigarette smoke. For instance, recent studies demonstrate that the reduction achieved from WHO-9 closely matches the reductions obtained from the average of WHO-39, FDA 18, the IARC list, etc., with only a 1 to 5% difference (Gunduz 2024).

In addition to WHO-9 list, NO and NOx can also be measured to test and confirm absence of combustion as defined in BSI PAS 8550: 2020 (BSI 2020). This publicly available specification sets the minimum standards for the quality, safety and performance of HTPs. It mandates testing for CO, NO, and NOx in order to verify the absence of combustion. Additional studies, including *in vitro* or *in silico* assessments or non-targeted chemical assessments, may provide further reassurance of appropriate product characterization if changes related to unknown flavor mixtures, botanical extract or new tobacco blend component.

3. Product use behavior

Whereas for vitamin C and plant stanols, it is possible to define intake levels appropriate for the claim, it is more difficult to define use behavior for HTPs. Establishing conditions of use for HTPs requires reassurance that consumers will use the products in such a way that they will be exposed to less HPHCs than if they remained using their conventional products. There are several elements that might affect the usage patterns for these products. Design changes may result in an increased use. Substantial changes in flavor or sensory aspects of the products may be useful to broaden the range of smokers who are successful in switching to HTPs, but it will be important to establish that they are not attracting non-smokers, nor are they resulting in substantially increased numbers of product units being used to such an extent that the achievement of reduced levels of HPHCs is nullified. Similarly, nicotine levels may have an influence on the way products are used which might result in different effective exposures to HPHCs from the reference product.

This stage of the bridging assessment aims to establish that consumer usage of the new product variant is consistent with the use patterns for the reference product:

- Pharmacokinetic testing for design changes that could affect exposures should be considered. Such changes might include a change in nicotine levels per unit; changes in physical aerosol characteristics; or substantial changes in flavor or sensory attributes. These changes will have been identified in the earlier characterization phase of the assessment.
- Abuse liability studies should establish that the product design does not result in different consumption behaviors (such as increased inhalation or number of product units) that might negate any advantages of reduced exposures or increase risk through increased uptake.
- Studies on perception and behavior for the product offering, (which includes both the new product design and any claims and marketing material associated with it), can be used to verify that the new product variant is clearly targeted at the appropriate consumer, (i.e., current smokers).

4. Post-marketing surveillance

Finally, it should be clear that post-marketing surveillance for the new product should be maintained in the same way that would have been established for the reference product.

## Summary & conclusions

Many consumer products claiming to help improve health or fitness are now very popular. This leads to common concerns around the marketing of such products, particularly given the health or harm reduction claims associated with the products, and the potential impact on consumers’ perception and behavior regarding such products. In parallel, there is a clear recognition that health claims are an important communication tool to inform and direct consumer choices to improve their personal health management (Ashwell et al 2023; Crawford et al 2022; European Union 2006; Lam et al 2022; Lenssen et al 2022; Sato et al 2023).

It is interesting to compare the situation for regulating health claims relating to dietary supplements or nutraceuticals to the harm reduction potential of HTPs. There is very little agreement internationally on the regulation of these products even though they are all aimed at providing consumer options for harm reduction (Ashwell et al 2023). The evolution of different tobacco regulations worldwide has resulted in varying regulatory environments for HTPs, with approaches for risk- or exposure-reduction claims only formally being authorized in the USA (FDA 2012a) and now in Greece (Government Gazette Issue Greece 2022). Nevertheless, where regulated, most countries recognize that the marketing of HTPs is only authorized on the understanding that there is a likely population benefit based on the potential harm reduction compared to continued smoking (Institute for Global Tobacco Control 2020). In contrast, health claims for dietary supplements and functional foods have some basis in regulations in many countries. Again, the underlying principle in this case is that it is beneficial for the population to be informed of the potential health benefits of these products (Ashwell et al 2023; Sato et al 2023).

The regulatory status for dietary supplements and nutraceuticals is considerably more mature than that for novel tobacco products, so opportunities to learn from the experience of those regulations are clear. For dietary supplements and nutraceuticals, the first stage in approving a product for the market is to ensure that the product is well-characterized and meets food safety and hygiene standards. Although these regulations do not specifically refer to bridging or comparability testing, the concept that is applied is consistent with the bridging concept that is the subject of this paper. The authorization to use a specific claim for a dietary supplement or nutraceutical is based on a review of the scientific evidence linking the well-characterized element with a potential health benefit. Therefore, once a claim is characterized and authorized, any manufacturer may apply the same claim to any product through what is effectively a bridging approach ensuring that the characterization criteria established for the claim is applicable for their new product (Ashwell et al 2023; Burdock 2024; Crawford et al 2022; Djaoudene et al 2023; EFSA 2011; Komala et al 2023; Lenssen et al 2022).

Looking at the case for biosimilars provides some insights into situations where there may be variability in a complex product due to differences in manufacturing and production in different iterations. The regulatory approach here has been to ensure the quality, biological activity, and safety of the biosimilars followed by a tiered approach for shortened approval based on the extent of the differences from the reference product (Declerck and Farouk Rezk 2017; Gutka et al 2018; Machado et al 2023).

A similar approach for HTPs is proposed here, whereby the nature of the HTP is characterized based on the key factors contributing to its potential harm reduction potential. Thus, given the weight of scientific evidence linking the absence of combustion to reduced levels of HPHCs in the aerosol, which leads to similar reductions in the causal chain of events leading to disease (Schlage et al 2020), it could be argued that any product that can be confidently characterized as an HTP can be acceptably marketed as such. Set of recommendations for bridging studies that can be used for HTPs includes, characterization, scientific substantiation of product claims, product use behavior, and post-marketing survey. Simple bridging or comparability studies should be able to characterize new HTPs sufficiently to demonstrate that product have comparable aerosol and risk profile compared to a predicate product that is already in the market to provide reassurance in the appropriate marketing of them. Although the best way for people who smoke to reduce the adverse health consequences of smoking is to quit, many find it difficult or are unwilling to stop smoking (Douglas et al 2018; Tattan-Birch et al 2022). Bridging approach proposed in this paper would enable innovation to put product in the market with speed and provide an acceptable alternative for adults who would otherwise continue to smoke.

## Abbreviations

CELSD: Chain of events linking smoking to disease
EFSA: European Food Safety Authority
FDA: U.S. Food and Drug Administration
HPHCs: Harmful and potentially harmful constituents
HTPs: Heated tobacco products
MRTP: Modified risk tobacco product
NDA: EFSA Panel on Nutrition, Novel Foods and Food Allergens
